# Supplementary material for: Parallel fast and slow recurrent cortical processing mediates target and distractor selection in visual search
Source: Commun Biol. 2020 Nov 19;3:689. doi: 10.1038/s42003-020-01423-0 (PMC7677324; doi:10.1038/s42003-020-01423-0)
Supplement: Supplementary file 1 — Supplementary Information [file 42003_2020_1423_MOESM1_ESM.pdf]

Supplementary materials

Parallel recurrent cortical processing mediates target and distractor selection in visual search (S.E. Donohue, M.A. Schoenfeld & J.-M. Hopf)

ERMF topography and source localization of the N1pc

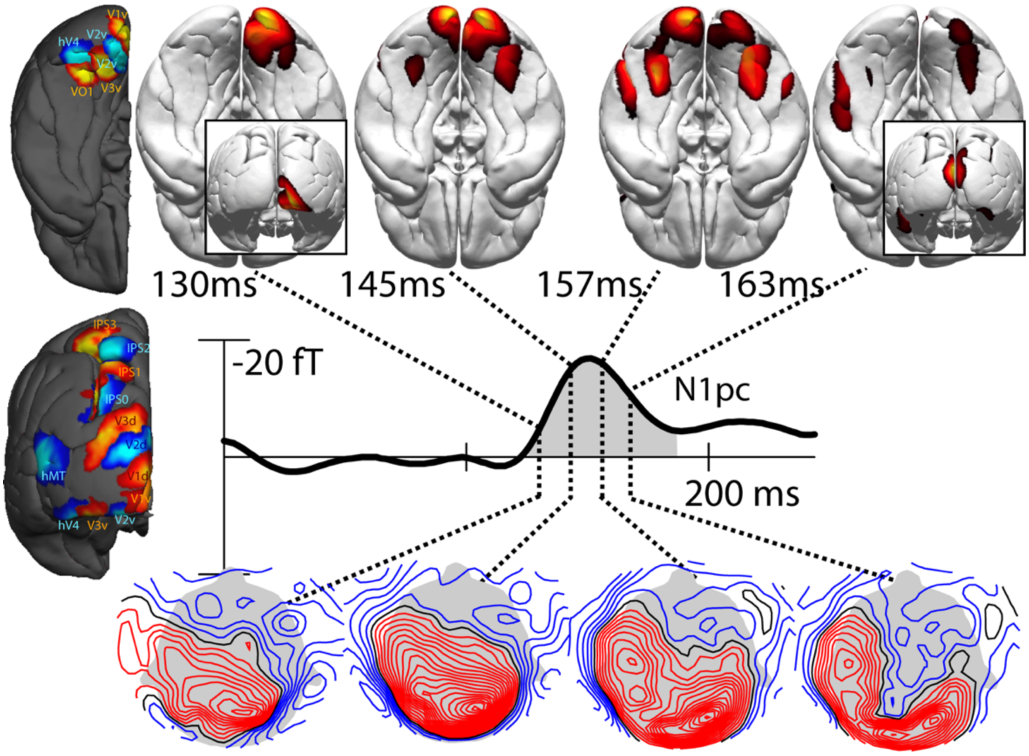

Supplementary Figure 1.

Shown are event-related magnetic field (ERMF) distributions and CSD estimates of the N1pc at selected time points after search frame onset. The data were collapsed over the Nt and Pd difference. Source activity underlying the N1pc starts in early visual cortex from where it propagates towards higher-tier anterior-lateral areas in ventral extrastriate cortex. By ~160ms source activity reappears in early visual cortex (inset). In addition, source activity in parietal cortex areas starts to appear. Parts of the N1pc arise from ventral extrastriate and parietal cortical areas which also generate the Nt and Pd. However, while the Nt and Pd represent recurrent modulations propagating from higher to lower levels in visual cortex, the N1pc is initially a forward-propagating modulation in those visual areas starting in

primary visual cortex. An illustration of the propagation of source activity underlying the N1pc is provided in Supplementary movie 3.

The Post-Nt component.

The tERL, but not the dERL (Figure 2), shows a modulation following the Nt (post-Nt) in a time range between 350-500 ms. Negative modulations following the N2pc have been described previously [1–7], which likely represent the sustained posterior contralateral negativity (SPCN). The SPCN, which is equivalent to at least early portions of the contralateral delay activity (CDA) [8,9], is a component indexing the maintenance of task relevant information in visual working memory (VWM). The present search experiment does not require the maintenance of target information over second-long delay periods, that a typical CDA experiment would require. However, the SPCN has been suggested to index the maintenance of target information as a working representation, to enable further computations aiding in target discrimination [2,3,5,6]. This is particularly relevant when target selection requires discrimination rather than simple identification. In fact, the present task requires orientation discrimination of the target following its localization based on color, which would be consistent with the need for a working representation of the target. It would also align with the fact that there is no such modulation for the distractor – an argument in favor of an SPCN interpretation put forward previously [3]. The current sources generating the post-Nt originate and are static in mid-level extrastriate areas (V2/3), which is compatible with the upholding of a sensory representation of discrimination-relevant information for the target in these visual areas. The present data, of course, cannot ultimately determine the exact nature of the post-Nt modulation, which requires more experimental work.

References

1. Wauschkuhn, B. *et al.* Lateralized human cortical activity for shifting visuospatial attention and initiating saccades. *J. Neurophysiol.* **80**, 2900–2910 (1998).
2. Mazza, V., Turatto, M., Umilta, C. & Eimer, M. Attentional selection and identification of visual objects are reflected by distinct electrophysiological responses. *Exp Brain Res* **181**, 531–6 (2007).
3. Jolicoeur, P., Brisson, B. & Robitaille, N. Dissociation of the N2pc and sustained posterior contralateral negativity in a choice response task. *Brain Res* **1215**, 160–72 (2008).

4. Corriveau, I. *et al.* Electrophysiological evidence of multitasking impairment of attentional deployment reflects target-specific processing, not distractor inhibition. *Int J Psychophysiol* **86**, 152–159 (2012).
5. Jannati, A., Gaspar, J. M. & McDonald, J. J. Tracking target and distractor processing in fixed-feature visual search: evidence from human electrophysiology. *J Exp Psychol Hum Percept Perform* **39**, 1713–30 (2013).
6. McDonald, J. J., Green, J. J., Jannati, A. & Di Lollo, V. On the electrophysiological evidence for the capture of visual attention. *J Exp Psychol Hum Percept Perform* **39**, 849–60 (2013).
7. Gaspar, J. M. & McDonald, J. J. Suppression of salient objects prevents distraction in visual search. *J Neurosci* **34**, 5658–66 (2014).
8. Vogel, E. K. & Machizawa, M. G. Neural activity predicts individual differences in visual working memory capacity. *Nature* **428**, 748–51 (2004).
9. Vogel, E. K., McCollough, A. W. & Machizawa, M. G. Neural measures reveal individual differences in controlling access to working memory. *Nature* **438**, 500–3 (2005).
