# Supplementary material for: Parallel fast and slow recurrent cortical processing mediates target and distractor selection in visual search
Source: Commun Biol. 2020 Nov 19;3:689. doi: 10.1038/s42003-020-01423-0 (PMC7677324; doi:10.1038/s42003-020-01423-0)
Supplement: Supplementary file 3 — Description of Additional Supplementary Files [file 42003_2020_1423_MOESM3_ESM.pdf]

Description of Additional Supplementary Files

File Name: Supplementary Movie 1
Description: Movie illustrating the propagation of source activity in visual cortex as well as the corresponding change of the ERMF distribution underlying the Nt component.

File Name: Supplementary Movie 2
Description: Movie illustrating the propagation of source activity in visual cortex as well as the corresponding change of the ERMF distribution underlying the Pd component.

File Name: Supplementary Movie 3
Description: Movie illustrating the propagation of source activity in visual cortex as well as the corresponding change of the ERMF distribution underlying the N1pc component.

File Name: Supplementary Data 1
Description: Supplementary data for Figures 2, 3, 4, and 5.
