# Supplementary material for: Parallel fast and slow recurrent cortical processing mediates target and distractor selection in visual search
Source: Commun Biol. 2020 Nov 19;3:689. doi: 10.1038/s42003-020-01423-0 (PMC7677324; doi:10.1038/s42003-020-01423-0)
Supplement: Supplementary file 8 — Reporting Summary [file 42003_2020_1423_MOESM8_ESM.pdf]

nature research

Corresponding author(s): Jens-Max Hopf
Last updated by author(s): Sep 3, 2020

Reporting Summary

Nature Research wishes to improve the reproducibility of the work that we publish. This form provides structure for consistency and transparency in reporting. For further information on Nature Research policies, see our Editorial Policies and the Editorial Policy Checklist.

Statistics

For all statistical analyses, confirm that the following items are present in the figure legend, table legend, main text, or Methods section.

n/a	Confirmed	
☐	☒	The exact sample size (*n*) for each experimental group/condition, given as a discrete number and unit of measurement
☐	☒	A statement on whether measurements were taken from distinct samples or whether the same sample was measured repeatedly
☐	☒	The statistical test(s) used AND whether they are one- or two-sided<br>*Only common tests should be described solely by name; describe more complex techniques in the Methods section.*
☒	☐	A description of all covariates tested
☐	☒	A description of any assumptions or corrections, such as tests of normality and adjustment for multiple comparisons
☐	☒	A full description of the statistical parameters including central tendency (e.g. means) or other basic estimates (e.g. regression coefficient) AND variation (e.g. standard deviation) or associated estimates of uncertainty (e.g. confidence intervals)
☐	☒	For null hypothesis testing, the test statistic (e.g. *F*, *t*, *r*) with confidence intervals, effect sizes, degrees of freedom and *P* value noted<br>*Give P values as exact values whenever suitable.*
☒	☐	For Bayesian analysis, information on the choice of priors and Markov chain Monte Carlo settings
☒	☐	For hierarchical and complex designs, identification of the appropriate level for tests and full reporting of outcomes
☐	☒	Estimates of effect sizes (e.g. Cohen's *d*, Pearson's *r*), indicating how they were calculated

Our web collection on statistics for biologists contains articles on many of the points above.

Software and code

Policy information about availability of computer code

Data collection	MSI Software of the BTI Magnes 3600 recording system
Data analysis	MSI software (BTI), Multi-modal neuroimaging Sofware Curry 8 (Compumedics Neuroscan), Matlab R14b, FreeSurfer (version 5.1.)

For manuscripts utilizing custom algorithms or software that are central to the research but not yet described in published literature, software must be made available to editors and reviewers. We strongly encourage code deposition in a community repository (e.g. GitHub). See the Nature Research guidelines for submitting code & software for further information.

Data

Policy information about availability of data

All manuscripts must include a data availability statement. This statement should provide the following information, where applicable:

- Accession codes, unique identifiers, or web links for publicly available datasets
- A list of figures that have associated raw data
- A description of any restrictions on data availability

The datasets generated during and/or analysed during the current study are available from the corresponding author on reasonable request.

Field-specific reporting

Please select the one below that is the best fit for your research. If you are not sure, read the appropriate sections before making your selection.

☒ Life sciences ☐ Behavioural & social sciences ☐ Ecological, evolutionary & environmental sciences

For a reference copy of the document with all sections, see nature.com/documents/nr-reporting-summary-flat.pdf

Life sciences study design

All studies must disclose on these points even when the disclosure is negative.

Sample size	Sample size was determined based on standard sample size in MEG/EEG experiments which guarantees robust measurement of the N2pc response in visual search.
Data exclusions	Five subjects were excluded because 30% or more of their trials contained physiological artifacts (e.g., blinks, horizontal eye movements).
Replication	The Nt and Pd components identified in the reported MEG study were perfectly replicated in two more MEG experiments (cf. Donohue et al. 2018, JN)
Randomization	Randomization was not relevant because there were no experimental groups, i.e. all participants performed the same experimental protocol.
Blinding	Blinding was not relevant because there were no experimental groups.

Reporting for specific materials, systems and methods

We require information from authors about some types of materials, experimental systems and methods used in many studies. Here, indicate whether each material, system or method listed is relevant to your study. If you are not sure if a list item applies to your research, read the appropriate section before selecting a response.

Materials & experimental systems

n/a	Involved in the study	
☒	☐	Antibodies
☒	☐	Eukaryotic cell lines
☒	☐	Palaeontology and archaeology
☒	☐	Animals and other organisms
☐	☒	Human research participants
☒	☐	Clinical data
☒	☐	Dual use research of concern

Methods

n/a	Involved in the study	
☒	☐	ChIP-seq
☒	☐	Flow cytometry
☒	☐	MRI-based neuroimaging

Human research participants

Policy information about studies involving human research participants

Population characteristics	Twenty-four human participants were included in this study (10 male, ages: 22-34, mean = 25.4 years old, 1 left-handed).
Recruitment	Participants were randomly selected from a list of available participants (mostly students of the OvGU)
Ethics oversight	Ethics committee of the Otto-von-Guericke University, Magdeburg

Note that full information on the approval of the study protocol must also be provided in the manuscript.
